# Recent sarcopenia definitions—prevalence, agreement and mortality associations among men: Findings from population‐based cohorts

**DOI:** 10.1002/jcsm.13160

**Published:** 2023-01-05

**Authors:** Leo D. Westbury, Charlotte Beaudart, Olivier Bruyère, Jane A. Cauley, Peggy Cawthon, Alfonso J. Cruz‐Jentoft, Elizabeth M. Curtis, Kristine Ensrud, Roger A. Fielding, Helena Johansson, John A. Kanis, Magnus K. Karlsson, Nancy E. Lane, Laetitia Lengelé, Mattias Lorentzon, Eugene McCloskey, Dan Mellström, Anne B. Newman, Claes Ohlsson, Eric Orwoll, Jean‐Yves Reginster, Eva Ribom, Björn E. Rosengren, John T. Schousboe, Eric J. Shiroma, Nicholas C. Harvey, Elaine M. Dennison, Cyrus Cooper, the International Musculoskeletal Ageing Network

**Affiliations:** ^1^ MRC Lifecourse Epidemiology Centre University of Southampton Southampton UK; ^2^ WHO Collaborating Centre for Public Health Aspects of Musculoskeletal Health and Aging, Division of Public Health, Epidemiology and Health Economics University of Liège Liège Belgium; ^3^ Department of Epidemiology, School of Public Health University of Pittsburgh Pittsburgh PA USA; ^4^ Research Institute California Pacific Medical Center San Francisco CA USA; ^5^ Department of Epidemiology and Biostatistics University of California San Francisco CA USA; ^6^ Servicio de Geriatría Hospital Universitario Ramón y Cajal (IRYCIS) Madrid Spain; ^7^ Medicine and Epidemiology & Community Health University of Minnesota Minneapolis MN USA; ^8^ Center for Care Delivery and Outcomes Research, Minneapolis VA Health Care System Minneapolis MN USA; ^9^ Nutrition, Exercise Physiology, and Sarcopenia Laboratory, Jean Mayer USDA Human Nutrition Research Center on Aging Tufts University Boston MA USA; ^10^ Mary MacKillop Institute for Health Research Australian Catholic University Melbourne Australia; ^11^ Sahlgrenska Osteoporosis Centre, Institute of Medicine University of Gothenburg Gothenburg Sweden; ^12^ Centre for Metabolic Bone Diseases University of Sheffield Sheffield UK; ^13^ Clinical and Molecular Osteoporosis Research Unit, Department of Clinical Sciences Malmo, Lund University and Department of Orthopedics Skane University Hospital Malmo Sweden; ^14^ Division of Rheumatology, Department of Internal Medicine UC Davis Health Sacramento CA USA; ^15^ Geriatric Medicine, Institute of Medicine, Sahlgrenska Academy Sahlgrenska University Hospital Mölndal Sweden; ^16^ Centre for Integrated Research in Musculoskeletal Ageing (CIMA), Mellanby Centre for Bone Research University of Sheffield Sheffield UK; ^17^ Centre for Bone and Arthritis Research (CBAR), Sahlgrenska Academy University of Gothenburg Gothenburg Sweden; ^18^ Department of Internal Medicine and Clinical Nutrition, Institute of Medicine, Sahlgrenska Osteoporosis Centre, Centre for Bone and Arthritis Research at the Sahlgrenska Academy University of Gothenburg Gothenburg Sweden; ^19^ Region Västra Götaland Sahlgrenska University Hospital, Department of Drug Treatment Gothenburg Sweden; ^20^ Oregon Health & Science University Portland OR USA; ^21^ Department of Surgical Sciences University of Uppsala Uppsala Sweden; ^22^ Park Nicollet Clinic and HealthPartners Institute Bloomington MN USA; ^23^ University of Minnesota Minneapolis MN USA; ^24^ Laboratory of Epidemiology and Population Sciences, Intramural Research Program National Institute on Aging Baltimore MD USA; ^25^ NIHR Southampton Biomedical Research Centre University of Southampton and University Hospital Southampton NHS Foundation Trust Southampton UK; ^26^ Victoria University of Wellington Wellington New Zealand; ^27^ NIHR Oxford Biomedical Research Centre University of Oxford Oxford UK

**Keywords:** Epidemiology, Sarcopenia, Ageing, Mortality, Prevalence

## Abstract

**Background:**

The 2019 European Working Group on Sarcopenia in Older People (EWGSOP2) and the Sarcopenia Definitions and Outcomes Consortium (SDOC) have recently proposed sarcopenia definitions. However, comparisons of the performance of these approaches in terms of thresholds employed, concordance in individuals and prediction of important health‐related outcomes such as death are limited. We addressed this in a large multinational assembly of cohort studies that included information on lean mass, muscle strength, physical performance and health outcomes.

**Methods:**

White men from the Health Aging and Body Composition (Health ABC) Study, Osteoporotic Fractures in Men (MrOS) Study cohorts (Sweden, USA), the Hertfordshire Cohort Study (HCS) and the Sarcopenia and Physical impairment with advancing Age (SarcoPhAge) Study were analysed. Appendicular lean mass (ALM) was ascertained using DXA; muscle strength by grip dynamometry; and usual gait speed over courses of 2.4–6 m. Deaths were recorded and verified. Definitions of sarcopenia were as follows: EWGSOP2 (grip strength <27 kg and ALM index <7.0 kg/m^2^), SDOC (grip strength <35.5 kg and gait speed <0.8 m/s) and Modified SDOC (grip strength <35.5 kg and gait speed <1.0 m/s). Cohen's kappa statistic was used to assess agreement between original definitions (EWGSOP2 and SDOC). Presence versus absence of sarcopenia according to each definition in relation to mortality risk was examined using Cox regression with adjustment for age and weight; estimates were combined across cohorts using random‐effects meta‐analysis.

**Results:**

Mean (SD) age of participants (*n* = 9170) was 74.3 (4.9) years; 5929 participants died during a mean (SD) follow‐up of 12.1 (5.5) years. The proportion with sarcopenia according to each definition was EWGSOP2 (1.1%), SDOC (1.7%) and Modified SDOC (5.3%). Agreement was weak between EWGSOP2 and SDOC (κ = 0.17). Pooled hazard ratios (95% CI) for mortality for presence versus absence of each definition were EWGSOP2 [1.76 (1.42, 2.18), *I* ^2^: 0.0%]; SDOC [2.75 (2.28, 3.31), *I* ^2^: 0.0%]; and Modified SDOC [1.93 (1.54, 2.41), *I* ^2^: 58.3%].

**Conclusions:**

There was low prevalence and poor agreement among recent sarcopenia definitions in community‐dwelling cohorts of older white men. All indices of sarcopenia were associated with mortality. The strong relationship between sarcopenia and mortality, regardless of the definition, illustrates that identification of appropriate management and lifecourse intervention strategies for this condition is of paramount importance.

## Introduction

Sarcopenia is a condition characterized by the excessive loss of muscle mass and strength with age; it is associated with physical disability, mortality, considerable healthcare costs and significant loss of quality of life.^1^, ^2^ Since 2016, sarcopenia has been recognized as a defined condition according to the International Classification of Diseases, Clinical Modification.^3^ However, there is currently no consensus definition for making the diagnosis of sarcopenia.

Various algorithms for defining sarcopenia have been proposed. In 2010, the European Working Group on Sarcopenia in Older People (EWGSOP) defined sarcopenia as low lean mass and either low strength (grip strength) or function (gait speed).^4^ The revised definition in 2019 (EWGSOP2) regards strength, rather than lean mass, as the primary sarcopenia component and defines probable sarcopenia as having low strength; confirmed sarcopenia as having low strength and lean mass; and severe sarcopenia as having low strength, lean mass and impaired function.^5^ The Foundation for the National Institutes of Health (FNIH) Sarcopenia Project, which was data driven, defined sarcopenia in 2014 as having weak grip strength and low appendicular lean mass (ALM) adjusted for body mass index (BMI).^6^ An updated version of this definition was proposed in 2020 by the Sarcopenia Definitions and Outcomes Consortium (SDOC), which defines sarcopenia in terms of weak grip strength and slow gait speed.^7^ Both SDOC and EWGSOP2 reflect research over the previous decade showing the greater capacity of muscle strength and function in comparison with lean mass in predicting risk of adverse health outcomes.^7^, ^8^, ^9^

It is well established that the prevalence of sarcopenia varies depending on geographical region and the age, ethnicity and setting of the population sampled as well as the definition used.^10^ However, comparisons of recent definitions (EWGSOP2 and SDOC) in terms of thresholds employed, concordance in individuals and prediction of important health‐related outcomes such as death are limited. Furthermore, to our knowledge, no studies have examined these definitions in terms of their prevalence or relationship with mortality when the original thresholds were modified. Such knowledge may enhance these definitions by providing more clinically relevant thresholds. We addressed these research areas in a large multinational assembly of cohort studies comprising the Health Aging and Body Composition (Health ABC) Study (USA), Osteoporotic Fractures in Men (MrOS) Study cohorts (Sweden, USA), the Hertfordshire Cohort Study (HCS) (UK) and the Sarcopenia and Physical impairment with advancing Age (SarcoPhAge) Study (Belgium). Interpretation of the analyses was restricted to white men as these participants formed the vast majority of those in this assembly of cohort studies.

## Methods

### Cohort studies used for analysis

The Health ABC Study comprises 3075 US men and women, aged 70–79 years, who were recruited in 1997–1998.^11^ A random sample of white and all black Medicare beneficiaries from around Memphis and Pittsburgh was obtained. Sampled participants received a mailing followed by a telephone eligibility screen. Eligible participants were those reporting no difficulty in walking one‐quarter of a mile or climbing 10 stairs. Individuals with the following characteristics were excluded: clear cognitive impairment; inability to communicate with the interviewer; having a life‐threatening illness or difficulties with activities of daily living; requiring a walking aid; currently enrolled in a lifestyle intervention trial; or having an intention of moving outside the area within 3 years. Written informed consent was provided by all participants, and the study was approved by the institutional review boards at the University of Tennessee and the University of Pittsburgh.

MrOS US comprises 5994 men, aged 65–100 years, who were enrolled at six sites between March 2000 and April 2002^12^ using a variety of recruitment strategies. Common strategies included the use of voter registration and participant databases; mailings from the Department of Motor Vehicles; common seniors' newspaper features and advertisements; and targeted presentations. MrOS Sweden comprises 3014 men, aged 69–81 years, who were recruited from Gothenburg, Malmö and Uppsala using national population registers between October 2001 and December 2004.^13^ Participants needed to be able to walk without help and be without bilateral hip replacements to be eligible for MrOS Sweden or MrOS US. Self‐defined race and ethnicity were recorded. All participants gave written informed consent, and ethics committees and institutional review boards at each centre approved the study.

The HCS comprises 2997 men and women born in Hertfordshire from 1931 to 1939 and who still lived there in 1998–2004 when they attended a baseline home interview and research clinic for a detailed characterization of their health status; the study has been described in detail previously.^14^, ^15^ A subset of HCS participants who underwent whole body dual‐energy X‐ray absorptiometry (DXA) in 2011–2012 (*n* = 346) were analysed in this manuscript. The HCS baseline investigations had ethical approval from the Hertfordshire and Bedfordshire Local Research Ethics Committee, and all participants gave written informed consent; ethical approval was obtained for all HCS follow‐up studies.

The SarcoPhAge Study comprises 534 participants, aged 65 years or older, who were recruited from an outpatient clinic in Liège, Belgium, and by press advertisement between June 2013 and June 2014. Participants with an amputated limb or a BMI > 50 kg/m^2^ were excluded. All participants provided written informed consent, and the study was approved by the ethics committee of the University Teaching Hospital of Liège. Further details of this study have been published previously.^16^

### Ascertainment of participant characteristics

Height and weight were measured and used to derive BMI. ALM was ascertained using DXA; muscle strength by grip dynamometry; and customary gait speed was measured as a marker of mobility. ALM index was calculated by dividing ALM (kg) by height^2^ (m). Deaths were recorded and verified; mean (SD) follow‐up times (years) were as follows: Health ABC 11.6 (4.9), MrOS US 13.0 (5.7), MrOS Sweden 11.4 (4.8), HCS 6.4 (1.2), SarcoPhAge 4.4 (1.1). Further details on the ascertainment of this information in each cohort, including the procedures and measurement devices used, are provided in *Table* S1.

### Definitions of sarcopenia

Sarcopenia was defined according to EWGSOP2^5^ and SDOC.^7^ Modified thresholds for grip strength (use of the SDOC grip strength threshold of <35.5 kg in the EWGSOP2 definition as opposed to <27 kg) and gait speed (<1.0 m/s in the SDOC definition as opposed to <0.8 m/s) were also used in analyses. These sarcopenia definitions, along with the original and modified thresholds for the sarcopenia components, are presented in *Table* 1.

**Table 1:** Definitions of sarcopenia and thresholds used for each sarcopenia component

Definition	Algorithm	Original thresholds	Modified thresholds
2019 European Working Group on Sarcopenia in Older People (EWGSOP2)^5^	Low grip strength and ALM index	Grip strength: <27 kg (M), <16 kg (W) ALM index:<7.0 kg/m^2^ (M), <5.5 kg/m^2^ (W)	Grip strength: <35.5 kg (M) and <20 kg (W) ALM index:<7.0 kg/m^2^ (M), <5.5 kg/m^2^ (W)
Sarcopenia Definitions and Outcomes Consortium (SDOC)^7^	Low grip strength and gait speed	Grip strength: <35.5 kg (M) and <20 kg (W) Gait speed: <0.8 m/s	Grip strength: <35.5 kg (M) and <20 kg (W) Gait speed: <1.0 m/s

ALM, appendicular lean mass; M, men; W, women.

All main analyses were restricted to white men. EWGSOP2 also proposed thresholds for ALM [<20 kg (M), <15 kg (W)]; ALM index [ALM (kg) /height^2^(m)] was used for analysis instead of ALM to ensure that height was accounted for.

### Analytical cohort

There is evidence that components of sarcopenia and overall prevalence of sarcopenia vary according to ethnicity^17^, ^18^; to ensure comparability between cohorts, analyses were restricted to white participants in MrOS US and Health ABC (over 95% of participants were of white ethnicity in HCS, MrOS Sweden and SarcoPhAge). As 88% of the remaining participants were men, the sample for the main analysis was then additionally restricted to men with complete data regarding all variables used in analysis (*n* = 9170). A flow diagram for the analysis sample regarding each cohort is presented in *Figure* S1. Sex‐stratified sensitivity analyses were performed among all ethnicities as described below. Analyses were conducted using Stata, release 17.0.

### Statistical methods

Participant characteristics, including the proportion with sarcopenia according to each definition, were described using summary statistics among the entire sample and within each cohort. Cohen's kappa statistic was used to assess agreement between the original sarcopenia definitions (EWGSOP2 and SDOC). Original and modified thresholds for EWGSOP2 and SDOC components (low grip strength and gait speed) in relation to their distributions were examined using histograms. The prevalence of EWGSOP2 and SDOC components and definitions according to age bands was examined using original and modified thresholds. Finally, components and definitions (based on original and modified thresholds) were examined in relation to mortality risk in each cohort using Cox regression with adjustment for age and weight; estimates from each cohort were combined using random‐effects meta‐analysis to address the heterogeneity observed between cohorts, as reflected by high *I* ^2^ values for some exposures. These mortality associations were also compared between the following EWGSOP2 categories: probable (grip strength <27 kg), confirmed (grip strength <27 kg and ALM index <7.0 kg/m^2^) and severe (grip strength <27 kg, ALM index <7.0 kg/m^2^ and gait speed ≤0.8 m/s).

### Sensitivity analyses

The following analyses were repeated among men and women separately (all ethnicities included): construction of histograms to illustrate thresholds of sarcopenia components in relation to their distributions and examination of components and definitions (based on original and modified thresholds) in relation to mortality risk after adjustment for age and weight. Some thresholds for components differed between men and women as illustrated in *Table* 1.

For participants who did not have their gait speed assessed over 6 m (8 ft in HCS and 4 m in SarcoPhAge), prevalences of sarcopenia were re‐calculated when gait speeds in these two cohorts were converted to those expected over 6 m using previously published equations.^19^, ^20^ The results presented below are based on the raw gait speed values.

## Results

Participant characteristics of the whole sample of white men and within each cohort are presented in *Table* 2. Mean (SD) age of the analysis sample (*n* = 9170) was 74.3 (4.9) years. The proportion of participants with sarcopenia in the whole sample according to each definition was as follows: EWGSOP2 (1.1%), SDOC (1.7%), Modified EWGSOP2 (5.5%) and Modified SDOC (5.3%). Within each cohort, agreement was weak (data not shown) between EWGSOP2 and SDOC (κ = 0.0–0.4 depending on cohort and κ = 0.17 when cohorts were pooled). Overall, 64.7% of participants died during follow‐up [mean (SD) follow‐up time to death or until participants were censored was 12.1 (5.5) years]. Participant characteristics of each cohort, stratified by sex, are presented in *Table* S2.

**Table 2:** Participant characteristics among white men

Characteristic [mean (SD) or N(%)]	All cohorts (*n* = 9170)	Health ABC (*n* = 908)	MrOS US (*n* = 5064)	MrOS Sweden (*n* = 2851)	HCS (*n* = 157)	SarcoPhAge (*n* = 190)
Age (years)	74.3 (4.9)	74.4 (2.9)	73.8 (5.9)	74.9 (3.1)	75.3 (2.5)	73.6 (6.1)
Height (cm)	174.4 (6.6)	173.4 (6.2)	174.5 (6.6)	174.8 (6.5)	173.9 (6.2)	171.8 (6.3)
Weight (kg)	82.3 (12.6)	81.2 (12.3)	83.5 (12.9)	80.5 (11.8)	82.3 (11.7)	81.4 (15.0)
BMI (kg/m^2^)	27.0 (3.7)	27.0 (3.7)	27.4 (3.8)	26.3 (3.5)	27.2 (3.6)	27.5 (4.6)
ALM (kg)	24.2 (3.3)	23.2 (3.2)	24.3 (3.4)	24.2 (3.2)	24.3 (2.7)	23.4 (3.8)
ALM index (kg/m^2^)	7.9 (0.9)	7.7 (0.9)	8.0 (0.9)	7.9 (0.8)	8.0 (0.7)	7.9 (1.1)
Grip strength (kg)	41.7 (8.3)	39.5 (7.7)	41.6 (8.5)	43.0 (7.8)	37.2 (7.2)	39.0 (9.5)
Gait speed (m/s)	1.27 (0.25)	1.29 (0.23)	1.25 (0.23)	1.32 (0.25)	0.82 (0.18)	1.05 (0.29)
Original sarcopenia definitions						
EWGSOP2	105 (1.1%)	14 (1.5%)	69 (1.4%)	13 (0.5%)	0 (0.0%)	9 (4.7%)
SDOC	152 (1.7%)	3 (0.3%)	79 (1.6%)	28 (1.0%)	24 (15.3%)	18 (9.5%)
Modified sarcopenia definitions						
EWGSOP2	505 (5.5%)	73 (8.0%)	282 (5.6%)	123 (4.3%)	5 (3.2%)	22 (11.6%)
SDOC	487 (5.3%)	28 (3.1%)	283 (5.6%)	88 (3.1%)	52 (33.1%)	36 (18.9%)
Died during follow‐up	5929 (64.7%)	627 (69.1%)	3255 (64.3%)	1973 (69.2%)	28 (17.8%)	46 (24.2%)
Follow‐up time (years)	12.1 (5.5)	11.6 (4.9)	13.0 (5.7)	11.4 (4.8)	6.4 (1.2)	4.4 (1.1)

ALM, appendicular lean mass; EWGSOP2, 2019 European Working Group on Sarcopenia in Older People; HCS, Hertfordshire Cohort Study; Health ABC, Health, Aging and Body Composition Study; MrOS, Osteoporotic Fractures in Men Study; SarcoPhAge, Sarcopenia and Physical impairment with advancing Age Study; SDOC, Sarcopenia Definitions and Outcomes Consortium.

Thresholds for original and modified definitions are presented in *Table* 1.

**Figure 1:**
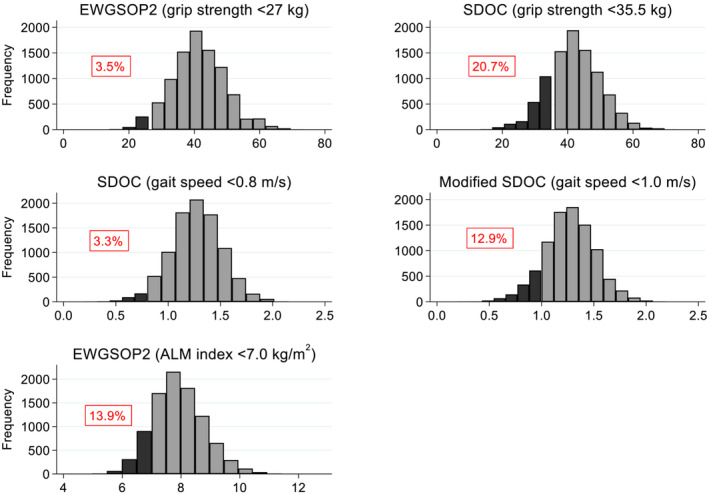
EWGSOP2 and SDOC thresholds for components in relation to their distributions among white men. ALM, appendicular lean mass; EWGSOP2, 2019 European Working Group on Sarcopenia in Older People (grip strength <27 kg; ALM index <7.0 kg/m^2^); SDOC, Sarcopenia Definitions and Outcomes Consortium (grip strength <35.5 kg; gait speed <0.8 m/s). Darker shading indicates values below the specified thresholds; the percentages below the thresholds are stated in each graph.

Histograms for grip strength, gait speed and ALM index are presented in *Figure* 1, with shading to indicate the proportion of values that were below various thresholds. A much higher proportion had low grip strength according to the SDOC threshold of <35.5 kg (20.7%) compared with the EWGSOP2 threshold of <27 kg (3.5%). When the SDOC gait speed threshold was increased from <0.8 to <1.0 m/s, the proportion with low gait speed increased considerably from 3.3% to 12.9%.

**Figure 2:**
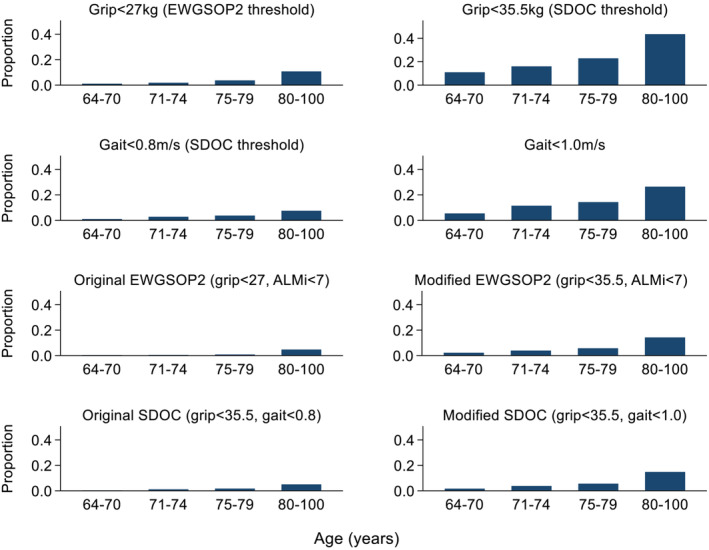
Prevalence of sarcopenia components and definitions according to age bands among white men. ALMi, appendicular lean mass index (kg/m^2^); EWGSOP2, 2019 European Working Group on Sarcopenia in Older People; SDOC, Sarcopenia Definitions and Outcomes Consortium.

The prevalence of sarcopenia components and definitions according to age bands is presented in *Figure* 2. For components and definitions using the SDOC grip strength threshold (<35.5 kg) and the Modified SDOC gait speed threshold (<1.0 m/s), generally, a steeper gradient of increased prevalence with advancing age was observed compared with other definitions using the EWGSOP2 grip strength threshold (<27 kg) and the original SDOC gait speed threshold (<0.8 m/s).

**Figure 3:**
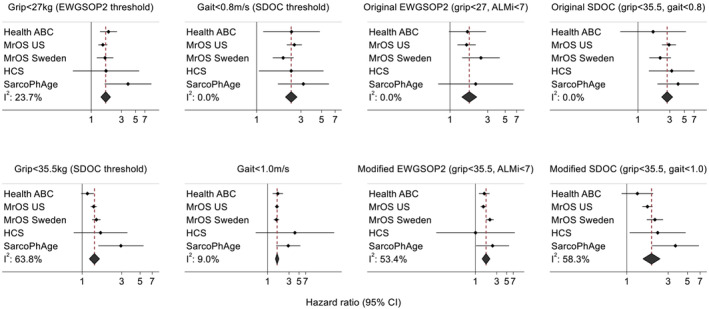
Original and modified EWGSOP2 and SDOC components and definitions in relation to risk of mortality among white men after adjustment for age and weight. ALMi, appendicular lean mass index (kg/m^2^); EWGSOP2, 2019 European Working Group on Sarcopenia in Older People; HCS, Hertfordshire Cohort Study; Health ABC, Health, Aging and Body Composition Study; MrOS, Osteoporotic Fractures in Men Study; SarcoPhAge, Sarcopenia and Physical impairment with advancing Age Study; SDOC, Sarcopenia Definitions and Outcomes Consortium. Estimates are missing for cohorts where no participants had the corresponding sarcopenia definition or component. Original EWGSOP2: grip strength <27 kg and ALM index <7.0 kg/m^2^; Modified EWGSOP2: grip strength <35.5 kg and ALM index <7.0 kg/m^2^. Original SDOC: grip strength <35.5 kg and gait speed <0.8 m/s; Modified SDOC: grip strength <35.5 kg and gait speed <1.0 m/s.

Hazard ratios for the presence versus absence of EWGSOP2 and SDOC components and definitions (original and modified) in relation to mortality risk, after adjustment for age and weight, are presented in *Figure* 3. Higher hazard ratios were observed for low grip strength and gait speed according to the original thresholds as these lower thresholds reflect poorer muscle strength and function. However, low grip strength and gait speed and the overall sarcopenia definitions were significantly associated with mortality risk regardless of whether the original or modified (less stringent) thresholds were used. For example, men meeting the Modified SDOC criteria for sarcopenia (grip strength <35.5 kg and gait speed <1.0 m/s) had a 1.9‐fold increase [hazard ratio (95% CI): 1.93 (1.54, 2.41), *I* ^2^: 58.3%] in risk of mortality compared with those without this condition.

**Figure 4:**
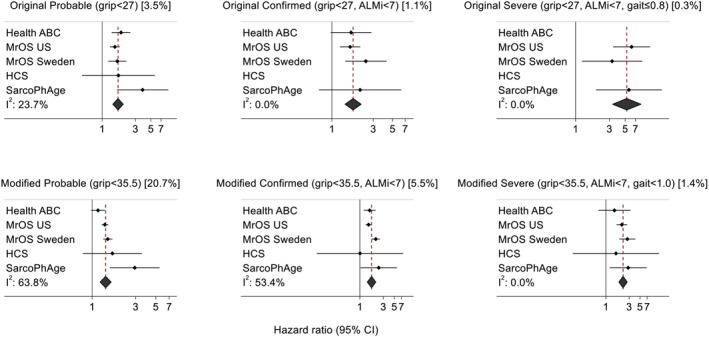
Original and modified EWGSOP2 definitions for probable, confirmed and severe sarcopenia in relation to risk of mortality among white men after adjustment for age and weight. ALMi, appendicular lean mass index (kg/m^2^); EWGSOP2, 2019 European Working Group on Sarcopenia in Older People; HCS, Hertfordshire Cohort Study; Health ABC, Health, Aging and Body Composition Study; MrOS, Osteoporotic Fractures in Men Study; SarcoPhAge, Sarcopenia and Physical impairment with advancing Age Study. Estimates are missing for cohorts where no participants had the corresponding sarcopenia definition. Original thresholds (graphs at the top of the figure): probable (grip strength <27 kg); confirmed (grip strength <27 kg and ALM index <7.0 kg/m^2^); and severe (grip strength <27 kg and ALM index <7.0 kg/m^2^ and gait speed ≤0.8 m/s). Modified thresholds for grip strength and gait speed are used in graphs at the bottom of the figure. Overall prevalence of the condition across all cohorts is stated in the graph subtitles in square brackets.

Progressively higher risks of mortality were observed for probable, confirmed and severe EWGSOP2 categories (*Figure* 4). This was the case with the original thresholds proposed and also when grip strength and gait speed thresholds were modified in the definitions to <35.5 kg and <1.0 m/s, respectively.

### Results from sensitivity analyses

The proportion with low grip strength and gait speed according to various thresholds was similar when men of all ethnicities were included (*Figure* S2); higher prevalences were observed among women for both gait speed thresholds and for the EWGSOP2 grip strength threshold (*Figure* S3). Similar patterns were observed regarding the mortality associations when participants of all ethnicities were included in sex‐stratified analysis (*Figures* S4 and S5); however, some of the associations among women did not reach statistical significance, possibly due to the smaller sample size.

When gait speed in HCS and SarcoPhAge was converted to 6 m, mean gait speed was higher, and prevalence of low gait speed and SDOC sarcopenia was lower in these cohorts compared to when the raw gait speed values were used. However, this resulted in minimal changes to the summary statistics for the overall analysis sample and no changes regarding findings on the association between sarcopenia definitions and risk of mortality (data not shown).

## Discussion

This study suggests a low prevalence of sarcopenia in relatively healthy community‐dwelling white men irrespective of the criteria used to define sarcopenia and establishes poor agreement between the EWGSOP2 and SDOC sarcopenia definitions as originally described. Furthermore, our analyses demonstrate the substantial differences in prevalence of sarcopenia that arise when different thresholds for grip strength and gait speed are adopted. Although thresholds can be selected through data‐driven approaches such as classification and regression tree analysis, the designation of thresholds of grip strength and gait speed used to identify individuals with sarcopenia is somewhat arbitrary, given the continuous distributions of these measures. As the prevalence of abnormality increases by moving the threshold towards the centre of the distribution, there will be a corresponding attenuation of the hazard ratio for clinical outcomes between individuals with and without the condition. Our data suggest that relatively modest alteration of these thresholds, such as using the modified versus original SDOC criteria, can deliver higher prevalence rates for sarcopenia in older population samples, while preserving the capacity to predict key health outcomes such as death. Key findings were similar in sensitivity analyses comprising men and women of all ethnicities. These findings, if replicated and validated, may contribute to the development of a global consensus on the definition of sarcopenia.

Thus, among these community‐dwelling cohorts of older men, the EWGSOP2 grip strength threshold (<27 kg) was at the 3.5th centile on the distribution, whereas the SDOC grip strength threshold (<35.5 kg) was at the 20.7th centile. With little effort, thresholds can be harmonised such that the SDOC grip strength threshold is used in the EWGSOP2 definition, and the International Working Group on Sarcopenia gait speed threshold (<1.0 m/s) is used in the SDOC definition. Adoption of these thresholds in the SDOC definition (grip strength <35.5 kg and gait speed <1.0 m/s) among white men in our study [mean (SD) age: 74.3 (4.9) years] led to a prevalence of sarcopenia of 5.3% and a hazard ratio (95% CI) for mortality of 1.93 (1.54, 2.41). In contrast, only 1.7% had SDOC sarcopenia according to original thresholds (grip strength <35.5 kg and gait speed <0.8 m/s).

It may be helpful to consider the prevalence of other non‐communicable disorders, the risk factors for which are continuously distributed in the general population. Osteoporosis (defined by the World Health Organization as a bone mineral density of at least 2.5 SDs below the young adult mean) had an average prevalence of 22.5% among women aged 50 years and over across France, Germany, Italy, Spain, the UK and Sweden in 2015.^21^ Hypercholesterolemia (total cholesterol ≥ 5.0 mmol/L or 190 mg/dL) had a global prevalence of 39% among adults in 2008.^22^ Hypertension (systolic blood pressure ≥140 mmHg, diastolic blood pressure ≥90 mmHg or use of antihypertensive medication) had a global prevalence of 34% among men and 32% among women, aged 30–79 years, in 2019.^23^ When placed in this context, it may be regarded as unusual to adopt thresholds for muscle strength and function that result in a definition of sarcopenia which only accommodates less than 2% of the population, even at ages above 70 years. Historically, thresholds used in definitions of some conditions, such as hypertension and hypercholesterolemia, closely reflect treatment thresholds. However, for sarcopenia, there is no presumption here that treatment decisions should be based on definitional approaches as there are likely to be different treatment thresholds depending on the feasibility, cost and efficacy of the interventions available. Therefore, cost‐effectiveness evaluation and other analyses are required to identify specific thresholds for identification of patients who would most likely benefit from treatments for sarcopenia.

Early sarcopenia definitions, such as those proposed in 1998 by Baumgartner^24^ and in 2007 by Delmonico,^25^ were based on lean mass. In 2010, the EWGSOP recognized that muscle strength does not only depend on lean mass and that the relationship between these quantities is non‐linear; they defined sarcopenia as having low lean mass with low strength or function.^4^ This was revised in 2019 (EWGSOP2) as research had established low strength as a stronger predictor of adverse outcomes than lean mass; low strength is the primary component in EWGSOP2 and is used alone to define probable sarcopenia with confirmed sarcopenia defined as having both low strength and lean mass.^5^ The EWGSOP2 approach aimed to promote diagnosis and management of sarcopenia in clinical practice, and, therefore, this definition aimed to identify indisputable cases.

In addition to the earlier sarcopenia definitions and those proposed by EWGSOP and EWGSOP2, FNIH and SDOC have also proposed definitions over the previous decade. The FNIH Sarcopenia Project researchers adopted the following approach in 2014: identified a grip strength threshold that discriminates mobility impairment (gait speed ≤0.8 m/s); identified ALM and ALM/BMI thresholds that discriminate this grip strength threshold; and examined the predictive capacity of these thresholds for incident mobility impairment and mortality.^6^ FNIH sarcopenia was characterized as having low grip strength and ALM/BMI. An updated version was proposed in 2020 by the SDOC.^7^ The SDOC used cohorts of community‐dwelling adults to identify thresholds for strength and lean mass parameters that discriminate low gait speed (<0.8 m/s) and then assessed predictive capacity of these thresholds for incident outcomes. SDOC sarcopenia was defined as having low grip strength and gait speed; lean mass was not consistently associated with outcomes. The differences between SDOC (analysis‐based approach) and EWGSOP2 (identification of indisputable clinical cases) may partly explain the lower grip strength threshold selected for the EWGSOP2 definition (<27 kg) compared with SDOC (<35.5 kg). In agreement with SDOC, the European Society for Clinical and Economic Aspects of Osteoporosis and Osteoarthritis (ESCEO) recommend that in clinical trials for drugs aimed at treating sarcopenia, cases should have a combination of low muscle strength and low physical performance.^26^

Few studies have compared the performance of EWGSOP2 and SDOC definitions in the same cohort. Their prevalence and association with fracture risk were compared in a study comprising the US, Sweden and Hong Kong MrOS cohorts.^27^ Similar to our findings regarding mortality, EWGSOP2 and SDOC were strongly associated with incident fracture (any, osteoporotic and major osteoporotic) and had low prevalence. However, in contrast to our study, low strength was characterized as having low grip strength or chair rise speed in the EWGSOP2 definition; this would result in a higher prevalence compared with the use of grip strength alone as a measure of strength. Prevalence of low grip strength using various thresholds was compared in a study comprising 98 men and women admitted to a geriatric rehabilitation hospital in Switzerland.^28^ As expected, prevalence of low grip strength according to the EWGSOP2 threshold (10.2%) was considerably lower compared with the SDOC threshold (19.4%). A previous SarcoPhAge analysis, using different components and thresholds compared with our study, reported similar effect sizes for EWGSOP2 and EWGSOP in relation to mortality; however, EWGSOP2 associations were not statistically significant due to its lower prevalence (7.4% vs 13.6%).^29^ Furthermore, severe EWGSOP2 was associated with a considerably greater risk of mortality compared to confirmed EWGSOP2, as demonstrated in our study.

Strengths of our study include the large number of participants the analyses were based on and that these individuals were recruited from established cohorts where data have been rigorously collected according to strict protocols. However, there are also several limitations of this study. First, the main analysis was only performed on white men; participants of the Health ABC cohort had no mobility disability at baseline; and MrOS participants had to be able to walk without the assistance of another person. These factors limit the generalizability of findings to the wider population of older people in this age range. Furthermore, the exclusion of participants with high risk of sarcopenia from this study, such as nursing home residents or those with advanced disability, suggests that the prevalence of sarcopenia in this age group across the general population may be much higher. However, ESCEO recommend that participants in clinical trials for drugs aimed at treating sarcopenia should be at least 70 years of age and that those who are severely malnourished or have extremely limited mobility should be excluded; such a population may be similar to that included in our study.^26^ Second, unlike approaches implemented in the FNIH Sarcopenia Project and SDOC to identify sarcopenia components and thresholds, only mortality and not incident disability was used as an outcome in this study. However, an advantage of using mortality as an outcome is that it is defined consistently across cohorts unlike incident disability, which may be defined differently across studies. Third, DXA lean mass includes muscle mass, organ weight, water and other non‐fat and non‐bone soft tissue and, therefore, is only a surrogate measure of muscle mass; previous studies suggest that other techniques, such as the D_3_‐creatine (D3‐Cr) dilution method, provide a more direct and accurate assessment of muscle mass, which is more strongly correlated with important clinical outcomes such as incident serious injurious falls, disability and mortality.^30^, ^31^, ^32^ Finally, some measurement protocols, such as distance covered during gait speed assessments, varied between cohorts. However, for participants who did not have their gait speed assessed over 6 m (8 ft in HCS and 4 m in SarcoPhAge), prevalences of sarcopenia in the pooled sample were similar when gait speeds in these two cohorts were converted to those expected over 6 m using previously published equations.^19^, ^20^ Although the lack of calibration of DXA and grip strength measures across cohorts may have affected the comparison of sarcopenia prevalence between cohorts, this is unlikely to have affected the mortality associations reported as Cox models were implemented internally within each cohort. For some exposures, high heterogeneity was observed between cohorts in the meta‐analysis. Possible reasons for this are that the eligibility criteria and geographical region differed between cohorts and the low prevalence of many exposures may have resulted in greater variability in estimates between cohorts.

## Conclusion

This study has examined the impact of raising the gait speed threshold in the SDOC algorithm from 0.8 to 1.0 m/s (sarcopenia characterized as grip strength <35.5 kg and gait speed <1.0 m/s). If one adopts this Modified SDOC approach, prevalence estimates for sarcopenia range from 3.1% to 5.6% in the cohorts enriched for healthy participation (Health ABC and MrOS) rising to 18.9% and 33.1% in those cohorts selected to represent the entire elderly population (SarcoPhAge and HCS, respectively). This definition has a higher prevalence among community‐dwelling older people, compared with the original SDOC definition, and remains strongly associated with mortality. These findings, if replicated and validated, will provide necessary insight about the appropriate prevalence of sarcopenia that a globally accepted definition of sarcopenia may adopt (contingent on cost‐effective analyses and other factors that would inform such a definition). This is an important consideration as a globally accepted definition of sarcopenia is required for large randomized controlled trials to evaluate efficacy and safety of interventions to prevent and treat sarcopenia.

## Conflict of interest

Cyrus Cooper reports personal fees (outside the submitted work) from Amgen, Danone, Eli Lilly, GSK, Kyowa Kirin, Medtronic, Merck, Nestle, Novartis, Pfizer, Roche, Servier, Shire, Takeda and UCB. Elaine Dennison has received lecture fees and honoraria from UCB, Pfizer, Lilly and Viatris outside of the submitted work. Nicholas Harvey reports consultancy, lecture fees and honoraria (outside the submitted work) from Alliance for Better Bone Health, AMGEN, MSD, Eli Lilly, Servier, Shire, UCB, Kyowa Kirin, Consilient Healthcare, Radius Health and Internis Pharma. Roger Fielding reports grants from the National Institutes of Health (National Institute on Aging) and the USDA, during the conduct of the study; grants, personal fees and other from Axcella Health; other from Inside Tracker; grants and personal fees from Biophytis; grants and personal fees from Astellas; personal fees from Pfizer; personal fees from Reneo; personal fees from Cytokinetics; personal fees from Amazentis; grants and personal fees from Nestle; and personal fees from Glaxo Smith Kline, outside the submitted work. Jean‐Yves Reginster declares grant support from industry through institution (IBSA‐Genevrier, Mylan, CNIEL, Radius Health, TRB), lecture fees when speaking at the invitation of sponsor [IBSA‐Genevrier, Mylan, CNIEL, Dairy Research Council (DRC), Nutricia, Danone, Agnovos] and consulting fees or paid advisory boards (IBSA‐Genevrier, Mylan, Radius Health, Pierre Fabre, Faes Pharma, Rejuvenate Biomed, Samumed, Teva, Theramex, Pfizer, Mithra Pharmaceuticals). The remaining authors declare that they have no conflicts of interest.

## Supporting information


**Table S1:** Ascertainment of participant information within each cohort
**Figure S1:** Flow diagram for the analysis sample regarding each cohort
**Table S2:** Participant characteristics according to cohort and sex
**Figure S2:** EWGSOP2 and SDOC thresholds for components in relation to their distributions among men from all ethnicities
**Figure S3:** EWGSOP2 and SDOC thresholds for components in relation to their distributions among women from all ethnicities
**Figure S4:** Original and modified EWGSOP2 and SDOC components and definitions in relation to risk of mortality among men from all ethnicities after adjustment for age and weight
**Figure S5:** Original and modified EWGSOP2 and SDOC components and definitions in relation to risk of mortality among women from all ethnicities after adjustment for age and weight
