# Supplementary material for: Recent sarcopenia definitions—prevalence, agreement and mortality associations among men: Findings from population‐based cohorts
Source: J Cachexia Sarcopenia Muscle. 2023 Jan 5;14(1):565–75. doi: 10.1002/jcsm.13160 (PMC9891989; doi:10.1002/jcsm.13160)
Supplement: Supplementary file 1 — Table S1: Ascertainment of participant information within each cohort Figure S1: Flow diagram for the analysis sample regarding each cohort Table S2: Participant characteristics according to cohort and sex Figure S2: EWGSOP2 and SDOC thresholds for components in relation to their distributions among men from all ethnicities Figure S3: EWGSOP2 and SDOC thresholds for components in relation to their distributions among women from all ethnicities Figure S4: Original and modified EWGSOP2 and SDOC components and definitions in relation to risk of mortality among men from all ethnicities after adjustment for age and weight Figure S5: Original and modified EWGSOP2 and SDOC components and definitions in relation to risk of mortality among women from all ethnicities after adjustment for age and weight [file JCSM-14-565-s001.docx]

**Article title:** Recent sarcopenia definitions - prevalence, agreement and mortality associations among men: findings from population-based cohorts

**Journal name:** The Journal of Cachexia, Sarcopenia and Muscle

**Author names:** Leo D Westbury, Charlotte Beaudart, Olivier Bruyère, Jane A Cauley, Peggy Cawthon, Alfonso J Cruz-Jentoft, Elizabeth M Curtis, Kristine Ensrud, Roger A Fielding, Helena Johansson, John A Kanis, Magnus K Karlsson, Nancy E Lane, Laetitia Lengelé, Mattias Lorentzon, Eugene McCloskey, Dan Mellström, Anne B Newman, Claes Ohlsson, Eric Orwoll, Jean-Yves Reginster, Eva Ribom, Björn E Rosengren, John T. Schousboe, Eric J Shiroma, Nicholas C Harvey, Elaine M Dennison, Cyrus Cooper and the International Musculoskeletal Ageing Network

**Affiliation and e-mail address of the corresponding author:**

Prof Cyrus Cooper

MRC Lifecourse Epidemiology Centre

University of Southampton

Southampton

UK

[cc@mrc.soton.ac.uk](mailto:cc@mrc.soton.ac.uk)

| **Table S1:** Ascertainment of participant information within each cohort | | | | | | |  |
| --- | --- | --- | --- | --- | --- | --- | --- |
|  |  |  |  | | |  |  |
| **Cohort** | **Height (cm)** | **Weight (kg)** | **Appendicular lean mass (kg)** | **Grip strength (kg)** | **Customary gait speed (m/s)** | | **Mortality** |
| Health, Aging and Body Composition Study | Measured using a Harpenden stadiometer | Measured using a standard balance beam scale | Ascertained from whole-body dual-energy X-ray absorptiometry scans (Hologic QDR 4500A; Hologic, Bedford, MA, USA) | Assessed twice for each hand using a Jamar dynamometer; the highest measurement was used for analysis. Participants with recent arthritis/pain in their wrist or hand or who had undergone surgery of the upper extremity in the past 3 months did not have their grip strength assessed on that side. | Calculated from the fastest time from two 6m gait speed tests. Participants were asked to walk at their usual pace. | | Deaths were determined from death certificates, hospital records and interviews with next of kin. All deaths were adjudicated by a central committee. |
| Osteoporotic Fractures in Men Study (US, Sweden) |  | Measured using an electric scale or balance beam scale | Ascertained from whole-body dual-energy X-ray absorptiometry scans.  MrOS US:  Hologic QDR 4500 [Hologic, Bedford, MA, USA]  MrOS Sweden:  Lunar Prodigy [GE Lunar Corp., Madison, WI, USA] |  |  |  | **Sweden:** Central registers covering all Swedish citizens were used to identify the date of death for all participants  **US:** Deaths were centrally adjudicated by a physician review of death certificates and hospital discharge summaries. |
| Hertfordshire Cohort Study |  | Measured using a SECA floor scale, Chasmors Ltd, London, UK | Ascertained from whole-body dual-energy X-ray absorptiometry scans (Lunar Prodigy Advanced Scanner, GE Medical Systems, UK) | Assessed three times for each hand using a Jamar dynamometer; the highest measurement was used for analysis | Calculated from the fastest time from two 2.44m (8ft) gait speed tests. Participants were asked to walk at their usual pace. | | This cohort was flagged on the NHS Central Register for continuous notification of deaths |
| Sarcopenia and Physical impairment with advancing Age Study | Measured using a rollable wall height meter | Measured using a floor scale | Ascertained from whole-body dual-energy X-ray absorptiometry scans (Hologic Discovery A, USA) | Assessed three times for each hand using a hydraulic dynamometer (Saehan Corporation, MSD Europe Bvba, Belgium); the highest measurement was used for analysis | Calculated from a 4m gait speed test. Participants were asked to walk at their usual pace. | | Mortality was collected annually by interview or phone call to relatives of participants and confirmed by medical records. |

**Figure S1:** Flow diagram for the analysis sample regarding each cohort


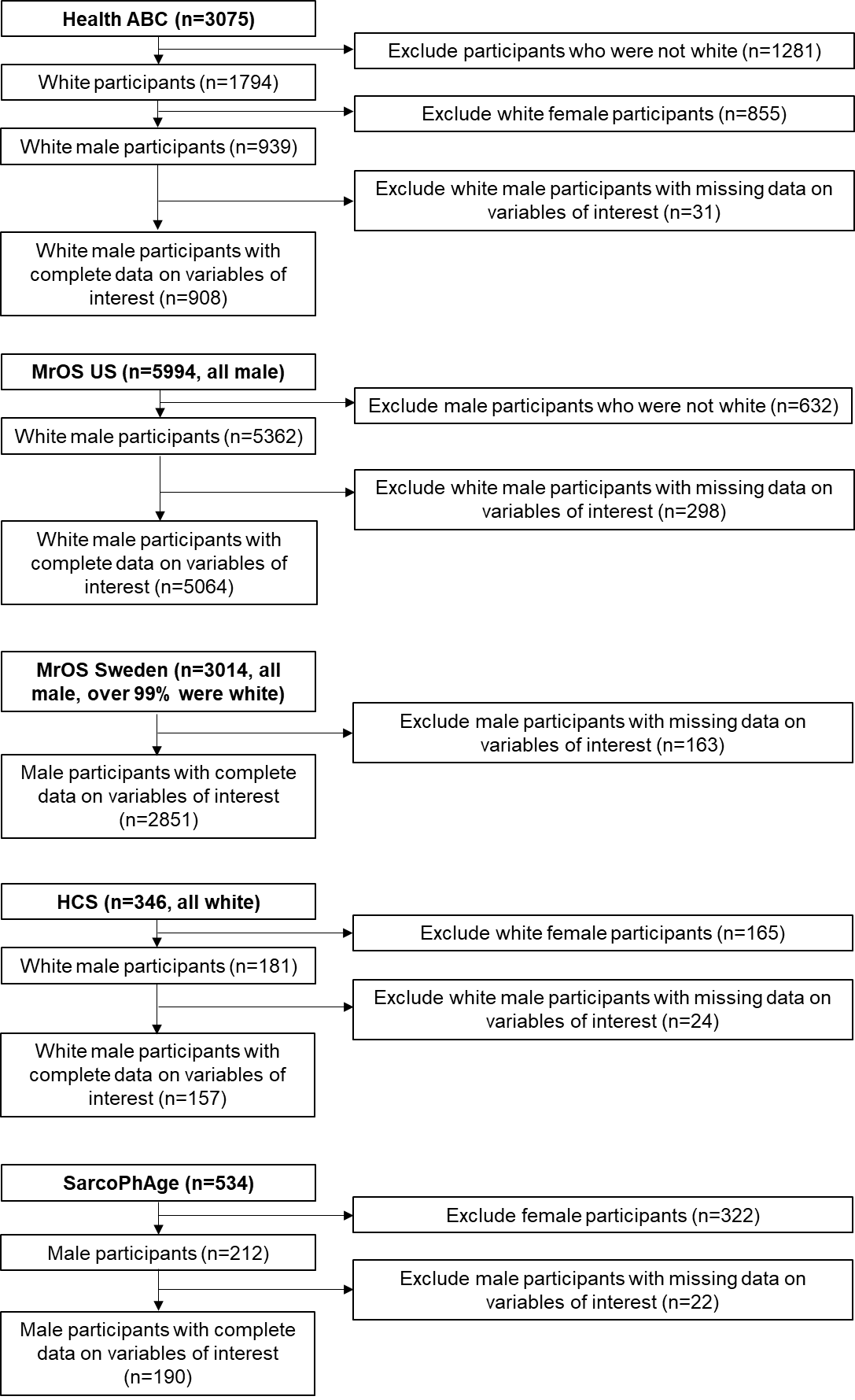


Variables of interest comprised those displayed in Table 2

HCS comprises 2997 men and women born in Hertfordshire from 1931-1939. 966 participants from East Hertfordshire underwent a dual-energy X-ray absorptiometry (DXA) BMD scan at baseline. In 2004, 642 were recruited to a musculoskeletal follow-up study. In 2011, 443/642 participated in a further follow-up study; 346/433 of these participants underwent whole body DXA.

SarcoPhAge participants were homogenous regarding ethnicity; the vast majority were white

| **Table S2: Participant characteristics according to cohort and sex** | | | | | | | | | |  |
| --- | --- | --- | --- | --- | --- | --- | --- | --- | --- | --- |
|  |  |  |  |  |  |  |  |  |  |  |
| **Characteristic [Mean (SD) or N(%)]** | **All cohorts (n=12238)** | **Health ABC (n=2949)** | | **MrOS US** | **MrOS Sweden** | **HCS (n=304)** | | **SarcoPhAge (n=472)** | |  |
|  |  | **Men (n=1428)** | **Women (n=1521)** | **Men (n=5662)** | **Men (n=2851)** | **Men (n=157)** | **Women (n=147)** | **Men (n=190)** | **Women (n=282)** |  |
| Ethnicity |  |  |  |  |  |  |  |  |  |  |
| White | 10425 (85.2%) | 908 (63.6%) | 826 (54.3%) | 5064 (89.4%) | 2851 (100.0%) | 157 (100.0%) | 147 (100.0%) | 190 (100.0%) | 282 (100.0%) |  |
| Black | 1444 (11.8%) | 520 (36.4%) | 695 (45.7%) | 229 (4.0%) | 0 (0.0%) | 0 (0.0%) | 0 (0.0%) | 0 (0.0%) | 0 (0.0%) |  |
| Asian | 181 (1.5%) | 0 (0.0%) | 0 (0.0%) | 181 (3.2%) | 0 (0.0%) | 0 (0.0%) | 0 (0.0%) | 0 (0.0%) | 0 (0.0%) |  |
| Hispanic | 120 (1.0%) | 0 (0.0%) | 0 (0.0%) | 120 (2.1%) | 0 (0.0%) | 0 (0.0%) | 0 (0.0%) | 0 (0.0%) | 0 (0.0%) |  |
| Other | 68 (0.6%) | 0 (0.0%) | 0 (0.0%) | 68 (1.2%) | 0 (0.0%) | 0 (0.0%) | 0 (0.0%) | 0 (0.0%) | 0 (0.0%) |  |
|  |  |  |  |  |  |  |  |  |  |  |
| Age (years) | 74.1 (4.7) | 74.3 (2.9) | 74.0 (2.9) | 73.7 (5.9) | 74.9 (3.1) | 75.3 (2.5) | 75.5 (2.6) | 73.6 (6.1) | 73.1 (5.9) |  |
| Height (cm) | 171.8 (8.5) | 173.2 (6.4) | 159.6 (6.1) | 174.2 (6.8) | 174.8 (6.5) | 173.9 (6.2) | 160.0 (5.6) | 171.8 (6.3) | 157.8 (6.7) |  |
| Weight (kg) | 80.1 (13.9) | 81.2 (13.1) | 70.4 (14.5) | 83.1 (13.2) | 80.5 (11.8) | 82.3 (11.7) | 70.9 (12.2) | 81.4 (15.0) | 64.8 (12.9) |  |
| BMI (kg/m^2^) | 27.1 (4.1) | 27.0 (3.9) | 27.7 (5.4) | 27.4 (3.8) | 26.3 (3.5) | 27.2 (3.6) | 27.7 (4.5) | 27.5 (4.6) | 26.0 (4.8) |  |
| ALM (kg) | 23.0 (4.4) | 23.9 (3.6) | 16.6 (3.2) | 24.3 (3.5) | 24.2 (3.2) | 24.3 (2.7) | 16.5 (2.1) | 23.4 (3.8) | 15.2 (2.7) |  |
| ALM index (kg/m^2^) | 7.7 (1.1) | 7.9 (1.0) | 6.5 (1.1) | 8.0 (0.9) | 7.9 (0.8) | 8.0 (0.7) | 6.4 (0.7) | 7.9 (1.1) | 6.1 (1.0) |  |
| Grip strength (kg) | 39.0 (10.3) | 40.6 (8.3) | 24.9 (5.8) | 41.6 (8.5) | 43.0 (7.8) | 37.2 (7.2) | 21.8 (6.1) | 39.0 (9.5) | 21.8 (6.0) |  |
| Gait speed (m/s) | 1.23 (0.26) | 1.24 (0.23) | 1.13 (0.22) | 1.25 (0.24) | 1.32 (0.25) | 0.82 (0.18) | 0.78 (0.19) | 1.05 (0.29) | 0.94 (0.27) |  |
|  |  |  |  |  |  |  |  |  |  |  |
| Original definitions |  |  |  |  |  |  |  |  |  |  |
| EWGSOP2 | 155 (1.3%) | 24 (1.7%) | 20 (1.3%) | 78 (1.4%) | 13 (0.5%) | 0 (0.0%) | 1 (0.7%) | 9 (4.7%) | 10 (3.5%) |  |
| SDOC | 284 (2.3%) | 14 (1.0%) | 18 (1.2%) | 103 (1.8%) | 28 (1.0%) | 24 (15.3%) | 28 (19.0%) | 18 (9.5%) | 51 (18.1%) |  |
|  |  |  |  |  |  |  |  |  |  |  |
| Modified definitions |  |  |  |  |  |  |  |  |  |  |
| EWGSOP2 | 662 (5.4%) | 97 (6.8%) | 56 (3.7%) | 324 (5.7%) | 123 (4.3%) | 5 (3.2%) | 3 (2.0%) | 22 (11.6%) | 32 (11.3%) |  |
| SDOC | 779 (6.4%) | 59 (4.1%) | 71 (4.7%) | 335 (5.9%) | 88 (3.1%) | 52 (33.1%) | 44 (29.9%) | 36 (18.9%) | 94 (33.3%) |  |
|  |  |  |  |  |  |  |  |  |  |  |
| Died during follow-up | 7566 (61.8%) | 1014 (71.0%) | 891 (58.6%) | 3582 (63.3%) | 1973 (69.2%) | 28 (17.8%) | 17 (11.6%) | 46 (24.2%) | 15 (5.3%) |  |
| Follow-up time (years) | 11.9 (5.5) | 11.2 (5.0) | 12.6 (4.6) | 13.0 (5.8) | 11.4 (4.8) | 6.4 (1.2) | 6.5 (1.0) | 4.4 (1.1) | 4.8 (0.5) |  |
| Health ABC: Health, Aging and Body Composition Study; MrOS: Osteoporotic Fractures in Men Study; HCS: Hertfordshire Cohort Study; SarcoPhAge: Sarcopenia and Physical impairment with advancing Age Study | | | | | | | | | |  |
|  |  |  |  |  |  |  |  |  |  |  |
| ALM: Appendicular lean mass | | | | | | | | | |  |
| EWGSOP2: 2019 European Working Group on Sarcopenia in Older People | | | | | | | | | |  |
| SDOC: Sarcopenia Definitions and Outcomes Consortium | | | | | | | | | |  |
| Thresholds for original and modified definitions are presented in Table 1 | | | | | | | | | |  |

**Figure S2:** EWGSOP2 and SDOC thresholds for components in relation to their distributions among men from all ethnicities

ALM: Appendicular lean mass

EWGSOP2: 2019 European Working Group on Sarcopenia in Older People (grip strength <27 kg; ALM index <7.0 kg/m^2^)

SDOC: Sarcopenia Definitions and Outcomes Consortium (grip strength <35.5 kg; gait speed <0.8 m/s)

Darker shading indicates values below the specified thresholds; the percentages below the thresholds are stated in each graph

**Figure S3:** EWGSOP2 and SDOC thresholds for components in relation to their distributions among women from all ethnicities

ALM: Appendicular lean mass

EWGSOP2: 2019 European Working Group on Sarcopenia in Older People (grip strength <16 kg; ALM index <5.5 kg/m^2^)

SDOC: Sarcopenia Definitions and Outcomes Consortium (grip strength <20 kg; gait speed <0.8 m/s)

Darker shading indicates values below the specified thresholds; the percentages below the thresholds are stated in each graph

**Figure S4:** Original and modified EWGSOP2 and SDOC components and definitions in relation to risk of mortality among men from all ethnicities after adjustment for age and weight

Health ABC: Health, Aging and Body Composition Study; MrOS: Osteoporotic Fractures in Men Study; HCS: Hertfordshire Cohort Study; SarcoPhAge: Sarcopenia and Physical impairment with advancing Age Study; ALMi: Appendicular lean mass index (kg/m^2^); EWGSOP2: 2019 European Working Group on Sarcopenia in Older People; SDOC: Sarcopenia Definitions and Outcomes Consortium

Original EWGSOP2: grip strength <27 kg and ALM index <7.0 kg/m^2^; Modified EWGSOP2: grip strength <35.5 kg and ALM index <7.0 kg/m^2^

Original SDOC: grip strength <35.5 kg and gait speed <0.8 m/s; Modified SDOC: grip strength <35.5 kg and gait speed <1.0 m/s

**Figure S5:** Original and modified EWGSOP2 and SDOC components and definitions in relation to risk of mortality among women from all ethnicities after adjustment for age and weight

Health ABC: Health, Aging and Body Composition Study; MrOS: Osteoporotic Fractures in Men Study; HCS: Hertfordshire Cohort Study; SarcoPhAge: Sarcopenia and Physical impairment with advancing Age Study; ALMi: Appendicular lean mass index (kg/m^2^); EWGSOP2: 2019 European Working Group on Sarcopenia in Older People; SDOC: Sarcopenia Definitions and Outcomes Consortium

Original EWGSOP2: grip strength <16 kg and ALM index <5.5 kg/m^2^; Modified EWGSOP2: grip strength <20 kg and ALM index <5.5 kg/m^2^

Original SDOC: grip strength <20 kg and gait speed <0.8 m/s; Modified SDOC: grip strength <20 kg and gait speed <1.0 m/s
